# UK Household Longitudinal Study: cohort profile

**DOI:** 10.1136/bmjmed-2025-002121

**Published:** 2026-09-18

**Authors:** Michaela Benzeval, Meena Kumari

**Affiliations:** 1 Institute for Social and Economic Research, University of Essex, Colchester, Essex CO4 3SQ, UK

**Keywords:** Socioeconomic Factors, Public health, Mental health, Genetics, Epidemiology

## Abstract

Understanding societal change and the consequences for different population groups is fundamental to social science and population health research. The UK Household Longitudinal Study is an infrastructure resource, in which data have been collected annually since 2009 from a sample of about 30 000 households selected to be representative of the four UK nations. Data are collected from all household members of all ages, and covers a wide range of topics, including health, biomarker, genetic, and epigenetic data and is freely available to researchers. The study has oversampled the five main ethnic minority groups in the UK to enable a sufficient sample size for analysis, and periodically boosts samples to ensure sufficient sample size and representativeness. The study incorporates and builds on the British Household Panel Survey; thus, data for a subsample of households are available back to 1991. The UK Household Longitudinal Study also has a smaller innovation panel with the same design that provides a platform for methodological research and innovation. Detailed social science information combined with multi-omics data, health survey data, and linkages to health and socioeconomic administrative data, enables research on the interaction between health, social, and economic change relevant to policymakers. The UK Household Longitudinal Study is widely used across biological, health, and social sciences: the study findings have been used extensively to influence social policy developments and public health.

**KEY MESSAGES**

- The UK Household Longitudinal Study is an extensive, nationally representative, and methodologically innovative study that follows participants and households over time
- Multidisciplinary and multitopic data collection, repeated annually, tracks changes in health over time and identifies how social and economic circumstances shape health trajectories
- The UK Household Longitudinal Study includes health assessments led by nurses that include a range of indicators (eg, blood pressure, lung function, grip strength, height, weight, waist circumference, and blood sampling) which enable risk factor analysis for common diseases and measure an array of additional factors, including measurement of genomic data
- Health assessments, multi-omic data, and repeated survey measures makes the UK Household Longitudinal Study a uniquely valuable resource for studying population health, social inequalities, and the influence of social environment and policy initiatives on biological and health outcomes

## Introduction

 The UK Household Longitudinal Study is an infrastructure resource established in 2009 as a nationally representative longitudinal study of households in the UK. The study is funded by the Economic and Social Research Council to allow free access to the data by the scientific and policy research communities.^1^ Information is collected annually across multiple domains for all household members on health, health behaviours and wellbeing, education, work and retirement, income and wealth, family relationships and much more. Additionally, the study included biological sample collection and clinical measures to provide objective health measures in adults. The survey enables research that considers policy questions on changes in health, behaviours, wellbeing, social mores, and social and economic structures. The study follows household members over time to explore the impact of the health and socioeconomic circumstances of one family member on other members’ health and circumstances, within the same household and when members move out to new homes.

### Study design and population characteristics

The fundamental design of the UK Household Longitudinal Study is an address based probability sample, so that the study is representative of household members across the UK (about 30 000 households, 48 000 adults, 5000 adolescents (10-15 years), and 11 000 children under 10 years at wave 1). Given the sample is based on known probabilities, biases created through non-response and attrition can be assessed, and the sample can be reweighted to be representative of the UK at baseline.^2^ Periodically, refreshment or boost samples are added to the panel to include immigrants who have moved to the UK since the baseline sample was taken, and to boost sample sizes. The study has two types of subsamples: general population samples based on probability sampling of households from clustered stratified postcode sectors recruited in 2009 (wave 1), boosted in 2022-24 (wave 14), and using the British Household Panel Study data originally sampled in 1991. Households in Northern Ireland were selected from a simple random sample. The second subsample comprises oversamples of the main five ethnicity minority groups in the UK at wave 1, repeated at wave 6 (2014-16) with the addition of new immigrant groups, and planned to be repeated for wave 20 (2028-30). Screening interviews were conducted at each baseline in areas where many people of ethnic minority groups live, to identify households comprising people from one of the identified ethnic groups, who are then recruited to the study.

People who join a study household (eg as study member partners, new babies, or lodgers) are temporarily included in the study while resident in the household. If these additional members have a child with an original sample member, or are born to a sample member, they become study members and are followed as such if they leave the original home. Any sample member who leaves their original household is followed, and the people with whom they form a new household become part of the study. Thus, the study follows general population dynamics, and maintains representativeness. A report on attrition is produced annually.

### Patient and public involvement

Since 2021, the study has recruited a participant panel that advises on study member engagement. The UK Household Longitudinal Study has a strategic oversight board that includes a range of stakeholders, including representatives from charities and public organisations, and academics from a wide range of disciplines.

### Data and measurements

The UK Household Longitudinal Study collects five types of data in the main study and in the innovation panel.

Firstly, questionnaire data, collected in different modes across a range of health domains relevant to different life stages (see table 1). From wave 1 to wave 7, a face-to-face survey was the main method of data collection, with minor mop-up surveys by telephone where face-to-face survey was not achieved. From wave 8, web data collection was also introduced, and the study became a mixed mode survey; thus, when the covid-19 pandemic started, the study was able to continue without any loss of fieldwork while undertaking nine additional COVID waves. This strategy includes annual measures of long term health conditions, and health scales such as the general health questionnaire (a widely used and validated measure of psychological distress), and the short form 12 (a validated measure of physical and mental health functioning). The study has rotating modules on health behaviours, sleep behaviours and wellbeing, and periodic measures of cognition (table 2) and psychological traits. Health measures for children are also collected, with information on maternal pregnancy and birth and specialist development scales at ages 1 year and 3 years of age (table 3). Parents complete the strengths and difficulties questionnaire about their children up to age 10 years. Once aged 10 years, children complete their own questionnaire that includes the strengths and difficulties questionnaire, as well as measures of wellbeing and health behaviours. Table 1 provides cross-sectional and longitudinal sample sizes for a few key measures.

**Table 1:** Sample sizes for illustrative health measures in the UK Household Longitudinal Study for adults aged ≥16 years, from waves 1 to 14 (2009-24)

Wave	Data collection period (calendar year)	No of adults with data available for each measure*				
		Has a longstanding health condition (main questionnaire, n=97 896)	GHQ† (self- complete, n=87 937)	Smoking status (n=80 053)	Alcohol consumption in last 12 months (from audit C‡, n=47 210)	Sleep quality (from Pittsburgh sleep questionnaire§, n=57 752)
Wave 1	2009-11	50 866	39 700	NA	NA	40 221
Wave 2	2010-12	54 520	43 423	50 664	NA	NA
Wave 3	2011-13	49 644	40 576	NA	NA	NA
Wave 4	2012-14	47 034	38 782	NA	NA	43 091
Wave 5	2013-15	44 808	37 136	40 967	NA	NA
Wave 6	2014-16	45 081	39 487	37 415	NA	NA
Wave 7	2015-17	42 081	37 452	39 303	37 643	39 266
Wave 8	2016-18	39 202	35 943	37 578	NA	NA
Wave 9	2017-19	36 001	33 265	34 935	33 509	NA
Wave 10	2018-20	34 218	31 899	33 471	NA	33 425
Wave 11	2019-21	31 894	30 341	31 483	30 728	NA
Wave 12	2020-22	29 151	31 899	28 427	18 948	NA
Wave 13	2021-23	27 872	27 133	27 836	27 518	27 790
Wave 14	2022-24	35 261	34 114	35 249	NA	NA
No of participants with at least three measures taken		60 694	53 791	46 152	29 801	28 342
No of participants measured at all possible waves		9095	6163	12 916	11 503	17 922

* Total number of unique contributors.

† Goldberg and Williams.

‡ Bush et al.

§ Buysse et al.

GHQ, general health questionnaire; NA, not applicable.

**Table 2:** Measurement and sample sizes for biological data collected in the UK Household Longitudinal Study waves 2 and 3 (2010-13)

Measure	Measurement instrument or method	No of participants with at least one successfully taken measure
Blood pressure and pulse	Measured three times on the right arm with the portable blood pressure monitor, the Omron HEM 907	20 410
Grip strength	Measured three times in the dominant hand with the Smedley's dynamometer	19 332
Standing height	Measured with a portable stadiometer	20 245
Lung function: peak expiratory flow, forced expiratory volume, forced vital capacity	Conducted with the electronic NDD Easy On-PC spirometer (in England and Wales) and the Vitalograph Escort (in Scotland)*	18 177
Waist circumference	Measured twice using a tape with an insertion buckle at one end	20 294
Weight and body fat percentage	Measured using a digital floor scale, the Tanita BF 522 scales	19 871
Albumin	From serum samples with a BCG colourimetric method on the Roche P module analyser.	12 948
Alkanine phosphatase	From serum samples with the International Federation of clinical chemistry (IFCC) colourimetric PNP method on the Roche P module analyser	12 812
Alanine transaminase	From serum samples with IFCC ultraviolet with pyridoxal phosphate activation method on the Roche P module analyser	12 804
Antibodies immunoglobulin G and M	From serum samples with an electrochemiluminsecent immunoassay on the Roche E170 analyser	12 924
Apolipoprotein A1	From serum sample using a nephelometric method on a BN II nephelometer (Siemens)	6310
Apolipoprotein B	From serum sample using a nephelometric method on a BN II nephelometer (Siemens)	6344
Apolipoprotein E	From serum sample using a nephelometric method on a BN II nephelometer (Siemens), using a non-isoform-specific polyclonal antibody	6340
Aspartate amino transferase	From serum samples with the IFCC ultraviolet with pyridoxal phosphase activation method on the Roche P module analyser	12 412
Total cholesterol	From serum on Roche Modular P analyser calibrated to the US Center for Disease Control guidelines	12 923
Creatinine	From serum samples using an enzymatic method on the Roche P module analyser.	12 946
C reactive protein	From serum with the N Latex CRP mono immunoassay on the Behring Nephelometer II Analyzer (Dade Behring)	12 558
Dihydroepiandrosterone	From serum samples on a competitive immunoassay on the Roche E module analyser	12 901
Factor H	From serum sample using a nephelometric method on a BN II nephelometer (Siemens)	3603
Ferritin	From serum samples by an electrochemiluminescent immunoassay on the Roche Modular E170 analyser	12 922
Fibrinogen	From citrate plasma with a modification of the Clauss thrombin clotting method on the IL-ACS-TOPS analyser	12 865
Gamma glutamyl transferase	From serum samples with an enzymatic method on the Roche P module analyser	12 844
Glycated Haemoglobin	From whole blood using the HPLC cation exchange on a Tosoh G8 analyser	12 189
Haemoglobin	From whole blood samples with a spectrophotometric assay on a Sysmex XE-2100 analyser	12 865
High density lipoprotein cholesterol	From serum on Roche Modular P analyser calibrated to the US Center for Disease Control guidelines	12 904
Insulin like growth factor 1	From serum samples by an electrochemiluminescent immunoassay on IDS ISYS analyser	12 859
Steroid hormone binding globulin	From serum samples by an electrochemiluminescence immunoassay on the Roche COBAS analyser	6288
Testosterone	From serum samples by an electrochemiluminescent immunoassay on the Roche Modular E170 analyser.	7850
Triglycerides	From serum on by the enzymatic method on a Roche P module analyser	12 926
Urea	From serum samples with a kinetic ultraviolet assay on a Roche P module analyser.	12 951
Proteomics	Olink Target 96 neurology panel and cardiometabolic panel	6180
Genotyping	Sincle nucleotide polymorphisms with Illumina HumanCoreExome BeadChip Kit (12v1-0) using DNA from people of white European descent†	9920
Epigenetics	Methylation with Illumina EPIC array using DNA from people of white European descent‡	3654
Cognition (wave 3, 2011-13)		
Memory	Perceived or self-rated memory	45 698
	Immediate word recall	45 679
	Delayed word recall	44 559
Executive function	Serial 7 subtraction	43 879
	Number series§	42 126
	Verbal fluency (number of animals in 1 minute)	45 081
	Numeric ability	44 941

* Scripting error in set 2 (see McFall 2013 for further details)

† There is no equivalence between the machines, so they should not be analysed together.

‡ More details available in Prim et al, 2017.

§ More details available in Bao et al, 2022.

**Table 3:** Sample sizes for illustrative health measures in the UK Household Longitudinal Study, asked at specific child ages, waves 1 to 14 (2009-24)

Measure	No of participants
**Reported by parent (usually mother)** *	
Birth weight	14 032
Behavioural difficulties (sleeping, crying, feeding) under 12 months of age	5268
Child has longstanding health condition at age 3 years	6351
Child has long standinghealth condition at age 5 years	6666
Child has long standinghealth condition at age 8 years	6992
SDQ† total difficulties score age 5 years	6405
SDQ† total difficulties score age 8 years	6752
**Self-report**	
SDQ† total difficulties score measured every other year between age 10-15 years:	
At least one measure taken	13 579
At least three measures taken	2621
Cognition (9 items‡ from Ravens Standard Progressive Matrices, wave 10 only)	2250-2460§

* For further details see: Institute for Social and Economic Research.

† Goodman R, Meltzer H, Bailey V.

‡ Bilker et al.

§ The number of participants for each item varies within this range.

Data regarding a wide range of other domains (eg, ethnicity, sexual orientation, geographical location, socioeconomic level, age) that could be relevant to population groups of interest, or act as explanatory factors or consequences in models of health (eg, education, employment status, income, housing, family relations), are also collected. A broader selection of geographical identifiers allowing linkage to contextual information (eg, index of multiple deprivation) are also provided in the study. In 2020 and 2021, pandemic related information was collected in April, May, June, July, September, November of 2020 and January and March of 2021.

The second data type collected is biomeasure data or marker data, gathered by nurses at waves 2 or 3. Table 2 lists the measures available, how they were measured and sample sizes achieved. In our innovation panel, we conduct a range of experiments relevant to the collection of health measures. During the 12th wave (in 2019), a range of experiments were conducted in biomarker data collection, led by participants.^3^ Additionally, viral serology is available from collection in April 2021.

We also collected measures of cognitive performance at wave 3 (table 3) and confirmed previously observed patterns of cognitive aging,^4^ and genomic data on a subsample of those who took part in the nurse interview and consented to DNA extraction^5^ (table 2).

Lastly, participants have also been asked to give consent to linkage of their data to routine health and other administrative data, with ongoing linkage processes (table 4). 

**Table 4:** Sample sizes for consent to data linkages in the UK Household Longitudinal Study, waves 1 to 14

Domain	Organisation supplying linkage data	Waves at which consent requested*	Linked sample size (No of participants)	Maximum consent*† (No of participants)	Access through
Health	NHS England‡	1, 4, COVID wave 8	8772	28 089	UK LLC
	NHS Scotland‡	1, 4, COVID wave 8	0	3304	UK LLC (to be confirmed)
	NHS Wales‡	1, 4, COVID wave 8	0	2479	UK LLC (forthcoming)
Education	National Pupil Database (England)	1,4,7,10,13	9378	10 736	UKDS
	Scottish Education	1,4,7,10,13	866	1062	UKDS
	Welsh Education	1,4,7,10,13	0	873	UK LLC (forthcoming)
Income	DWP	4,7,10,13	0	39 114	UK LLC (forthcoming)
	HMRC	5,8,11,14	0	36 129	UK LLC (forthcoming)
Personal finance	Credit histories	10, 13	18 868	19 052	UKDS
	NEST pensions§	11, 14	1672	17 691	UKDS
Environment	Energy use¶	8	12 501	17 991	ONS (forthcoming)
	Vehicle licence data**	5	16 947	18 580	UKDS

* The availability of linked data lags behind consent requests considerably; consent waves that have been linked linked are waves 1 (education, health), 4 (education, health), 5 (vehicle licence), 8 (energy use), 10 (credit history), 11 (National Employment Savings Trust), and COVID (health).

† Includes waves not yet matched, as well as those who consented to linkage but subsequently withdraw it or were not matchable. This includes people younger than 16 years for whom parents provide consent. If they do not give consent once they turn 16 years old, their data are not linked.

‡ Hospital episode statistics.

§ Many people consented to National Employment Savings Trust (NEST) linkage who did not have a NEST pension.

¶ Unit is number of addresses.

** Unit is number of vehicles.

DWP, Department for Work and Pensions; HMRC, HM Revenue & Customs; ONS, Office for National Statistics; UKDC, UK Data Service; UKLLC, UK Longitudinal Linkage Collaboration.

### Data availability

The UK Household Longitudinal Study data are freely available to researchers at the UK Data Service, with a new wave added each year. Most data are available through end user licence files and can be downloaded after registration. See box 1 for data references and box 2 for data user guides. Sensitive data (including geographic identifiers) are available by special licence or in the secure lab, which require an application process and for the secure data, specific training. Genomic data are available through the European Genome-Phenome Archive (EGAD00001016074; EGAD00001016073; EGAD00001016072) and combined with survey data by application. NHS and other administrative data linked to end user licence survey data are available through the UK Longitudinal Linkage Collaboration (table 4).

Box 1 **Key datasets availability and citations**

The bibliographic citations for key Understanding Society datasets:

- Main survey^30^
- Nurse collected clinical and biological data^31^
- Covid-19 survey^32^
- Innovation panel with methodological experiments^33^
- Linked NHS administrative data at UK Longitudinal Linkage Collaboration^34^

Information on other datasets from the UK Household Longitudinal Study can be found on the data releases website.^35^

Box 2 **Health relevant user guides in the UK Household Longitudinal Study**

**Main survey user guide**

The user guide contains information about the study, such as sample and questionnaire design, data collection and data processing, and information about using the dataset for research.^36^

**Pregnancy and early childhood (PEACH) dataset user guide**

This guide contains information about the pregnancy and early childhood data file, which collates this information into a single location to enable research into child development topics. ^27^

**Nurse health assessment user guide**

This guide describes the biomedical measures collected in waves 2 and 3 of the UK Household Longitudinal Study. A range of biomedical measures were collected during the health assessment, including blood pressure, weight, height, waist measurement, body fat composition, grip strength, and lung function.^26^

**Biomarker user guide and glossary**

The user guide contains information about the collection of biomarkers in UK Household Longitudinal Study. This document outlines the biomarkers currently available for study in the study, explores some of the factors to consider when using these biomarkers in analysis, and compares their prevalence with other key studies.^37^

**Proteomics user guide**

This guide describes the proteins available for analysis in UK Household Longitudinal Study.^38^

**Epigenetic ageing algorithms (epigenetic clocks) user guide**

This guide contains information about the five epigenetic clocks constructed in UK Household Longitudinal Study.^39^

**Polygenic scores user guide**

Polygenic scores are available in the special licence version of the nurse health assessment data. This guide contains information about polygenic scores and considerations when using them in analysis.^40^

**Cognitive ability measure**

This specialist guide describes the concepts, questions and measures for the cognitive ability modules included in wave 3 of the main and innovation panel surveys.^25^

User support can be found on the study website. ^41^

### Strengths and limitations

The unique design of the UK Household Longitudinal Study enables novel research that is generalisable to the entire population of the UK, and subgroups thereof, as well as considering relations between and within households and generations. The study also has sufficient sample size to investigate particular ethnic groups, and collects data from participants over an increasing portion of the lifespan as the panel lengthens. The study scope is across multiple domains including clinical, biomarker and omics data, and is strengthened by linkage to NHS administrative records.

Like all longitudinal studies, this study is susceptible to attrition, although the probability sample ensures that this can be documented and corrected for. Similarly, longitudinal studies struggle to balance the collection of consistent measures over time with capturing new research domains. The UK Household Longitudinal Study faces this dilemma, but the study team works with research communities to make effective choices on the content of the study. To date, biomarker data have been collected in one wave only, with genomic data limited to participants of white or European ancestry. However, both of these issues are to be resolved at wave 16 (see below).

### Key study findings

The UK Household Longitudinal Study is an infrastructure resource for the research community with users from a wide range of disciplines and sectors. The study team have identified over 4600 publications to date (August 2025) that have drawn on the data gathered in the UK Household Longitudinal Study. The study has contributed to a number of consortiums and genome wide association studies for phenotypes (eg, obesity,^6^ risk taking behaviours,^7^ and long covid^8^) or has acted as a control in case-control designs.^9^ Methylation data have been used in genome wide approaches; for example, to examine depression^10^ and to construct biological age algorithms for examining the association with social mobility^11^ and private renting.^12^

Using our biological data, research has shown that flexible working, in particular reduced hours working arrangements and allostatic load (a composite measure of physiologicalwear and tear associated with chronic stress) are connected.^13^ The longitudinal mental health data have been widely used in public health research, for example to describe the impact of lockdown conditions on mental health,^14^ the health and wellbeing before and after the transition into a young carer,^15^ and the effect of the hostile environment in England on the mental health of people in minority ethnic black groups.^16^ Data from the study have resulted in a number of publications describing social^17^ and place based differences in health measures.^18^

### Missing data

Despite the UK Household Longitudinal Study achieving response rates of around 90% for each wave, accumulating attrition can create biases in the study. ^19^ ^20^ However, given the sample is probability based, any biases from loss to follow-up can be identified and attenuated through the use of inverse probability weights (provided by the study) so population level inferences are possible. A second source of missing data is a result of the content rotation pattern used in the study to cover a broad range of domains while not overburdening participants. Table 1 shows that, despite the irregular timing of questions relevant to public health, the study still achieves substantial sample sizes for repeat measures over time.

### Future plans

The study is currently (2026) collecting data for waves 17 and 18. Wave 16 of the UK Household Longitudinal Study (2024-26) collected data across clinical and biological domains, including cognition,repeating those collected at waves 2 and 3 if possible. In addition, the collection of DNA is now being expanded to all adults, including those who are rising 16 (ie, participants who turned 16 years old since waves 2 and 3) to expand the genetic analysis of trios (two parents and a child) and within person analysis of change in DNA methylation. Gathered data will be made available in 2026 and 2027.^21^ The study continues to collect health data as described in table 1 at all waves. Funding is currently in place for wave 22 (2030-32).
