# Supplementary material for: UK Household Longitudinal Study: cohort profile
Source: BMJ Med. 2026 Sep 18;5(1):e002121. doi: 10.1136/bmjmed-2025-002121 (PMC13599898; doi:10.1136/bmjmed-2025-002121)
Supplement: Supplementary file 1 [file bmjmed-2025-002121.reviewer_comments.pdf]

PEER REVIEW HISTORY

BMJ Medicine publishes all reviews undertaken for accepted manuscripts. Reviewers are asked to complete a checklist review form and are provided with free text boxes to elaborate on their assessment. These free text comments are reproduced below.

ARTICLE DETAILS

**TITLE (PROVISIONAL)**	Understanding Society: UK Household Longitudinal Study (UKHLS), a resource for the public health research community
**AUTHORS**	Kumari, Meena; Benzeval, Michaela

VERSION 1 - REVIEW

**REVIEWER NAME**	Harron, Katie
**REVIEWER AFFILIATION**	
**REVIEWER CONFLICT OF INTEREST**	None
**AI DISCLOSURE**	No
**DATE REVIEW RETURNED**	06-Oct-2025

**GENERAL COMMENTS**	Overall I think this is a really informative and clearly written data resource profile. I just have a few minor comments.<br><br>What this data resource adds: This section says that for some households, data goes back to 1991. It would be helpful here to state when the main data collection began.<br><br>Table 1 – if they are currently on wave 16/17 data collection, why does this table not include wave 15? Do the numbers in this table represent unique individuals? If so, would it be possible to give the number of households too?<br><br>Could you expand a bit more on the dynamic nature of the cohort – and the number of households / individuals for whom you have data for the whole period? i.e. what is the average (and total) length of follow up within the study?<br><br>Would it be possible to include some simple characteristics of those included, e.g. age / sex / region / ethnicity?<br><br>Could you provide a bit more information about the involvement rates? i.e. for those who are invited, what percentage typically respond?

**REVIEWER NAME**	Davies, Neil
**REVIEWER AFFILIATION**	UCL, Division of Psychiatry and Department of Statistical Science
**REVIEWER CONFLICT OF INTEREST**	None
**AI DISCLOSURE**	No
**DATE REVIEW RETURNED**	08-Oct-2025

GENERAL COMMENTS	bmjmed-2025-002121: Understanding Society: UK Household Longitudinal Study (UKHLS), a resource for the public health research community<br><br>This paper nicely summarises the Understanding Society study, which is a flag ship investment from the ESRC and is very widely used across biomedical and social science. The paper is well written and would be useful for the research community. I have some suggestions below that I hope help improve the paper.<br><br>Section 1 – I would add genetic and epigenetic data here.<br>Section 3- It would be useful to provide some summary of the number of applications to use the data, or the number of papers published (if possible?)<br>“Findings from the UKHLS…” This needs references.<br><br>“Additionally, biosampling and clnical…” – would be good to summarise the ages or years when the biosampling occurred if possible here.<br><br>One of the unique features of US is that it is a representative random sample. Are there any key results or references that demonstrates the importance of this design for getting unbiased or less biased estimates? This is a key strength relative to other studies, such as the UK Biobank, that are bigger, but potentially more biased by differential ascertainment. You do make this point, but it really needs a compelling example to demonstrate how important this is for epidemiological studies. Alterantively, do we know when this is likely to be most, and less important (e.g. genetic studies, studies of inequalities, descriptive studies)? Feel free to say these are all unknowns that need more work. Do we have any information on the relative representativeness of US vs other representative samples such as the HSE? Is there code to create weights for analytic samples?<br><br>Linkage – US is also available within the LLC, might be worth flagging this in the penultimate para of page 7.<br><br>Genomic and Methylation data – would be worth including more details on this – how many samples, what chips, what imputation and QC have been done etc. This is another key feature of US, and would be great to include the relevant details for your readers.<br><br>Boxes 1 and 2 are useful – for typesetting it would be good to make sure the links are well formatted on the website etc.<br><br>Would it be worth including adding the genomics and methylation data to the LLC in the future plans?<br><br>Would it be possible to create a graph that summarises the data collection in US? For example, we used the following figure in our recent IJE profile on the genomics resources for the CLS cohorts (Figure 1):<br><br>https://academic.oup.com/ije/article/54/5/dyaf141/8237599<br><br>Are there any resources e.g. code for QC and data cleaning? Would it be worth flagging any of the work by CLOSER etc (totally optional, a reasonable response is “no”.

	Otherwise looks great, thanks for writing.

**REVIEWER NAME**	Islam, Nazrul
**REVIEWER AFFILIATION**	University of Southampton, School of Primary Care, Population Sciences and Medical Education
**REVIEWER CONFLICT OF INTEREST**	None
**AI DISCLOSURE**	No
**DATE REVIEW RETURNED**	10-Nov-2025

**GENERAL COMMENTS**	Thank you for the opportunity to review the manuscript which is written well. I hope the authors will find the following comments and suggestions useful for their revision.<br><br>"The fundamental design of UKHLS is an address-based…" does it mean postcode based?<br>"What does the UKHLS measure?" section says it has four types of data— I would suggest using sub-headlines with exact four types (currently more than four subsections).<br><br>"Measures of cognitive performance were collected at wave 3 (Table 3)…" It is not clear which cognitive measures are being referred to; table 3 only says "Cognition – 9 items from Ravens Standard Progressive Matrices (wave 10 only)".<br><br>"…confirm previously observed patterns of cognitive aging." Not sure what this is referring to.<br>"Genomic data on a sub-sample of those who took part in the nurse interview and consented to DNA extraction." Could the authors provide a bit more information on the response rate, any sampling bias, and any proposed measures to make them representative? If not, could be included as a limitation.<br><br>"Participants have also been asked to give consent to linkage of their data to routine health and other administrative data, with ongoing linkage processes (Table 4)." First, this should not be included under four types of data; maybe elsewhere. Second, could the authors clarify which datasets (primary care, secondary care etc) are these referring to?<br><br>A table showing the baseline characteristics of the original wave, most recent wave, and national population would be helpful to show representativeness.<br><br>A visual flow diagram showing how many participants continued to remain in the cohort, and how many participants were later included (and whether they remained or lost to follow-up) including their children could be very informative.<br><br>Minor: Some sources are referred to as hyperlinks. Perhaps better to use as proper sources using proper format?

**REVIEWER NAME**	Emmerson, Chris
**REVIEWER AFFILIATION**	Public Health Wales, Health Improvement Division
**REVIEWER CONFLICT OF INTEREST**	None

**AI DISCLOSURE**	No
**DATE REVIEW RETURNED**	23-Nov-2025

**GENERAL COMMENTS**	Overview<br>This paper describes the UK Household Longitudinal Study (UKHLS) to support public health researchers in understanding the data available within the study, its design and data collection methods and the research questions for which it can be used most effectively.<br>A brief review of peer-reviewed papers published in the BMJ and other journals widely used in the public health research community that report on research using the UKHLS suggests that this paper addresses a gap within the literature. In referencing their data source, these papers typically either refer to the UKHLS documentation and user guides (https://www.understandingsociety.ac.uk/about/about-the-study/), papers which are not peer reviewed, and do not necessarily provided a rounded view of the data set (e.g. Benzeval et al, 2023) or a paper (Buck and McFall, 2012) that is 13 years old.<br>Therefore a peer reviewed paper with up-to-date information for public health researchers on the UKHLS, as exist for other large datasets used in public health research (e.g. the 1970 British Cohort Study, Sullivan et al, 2023 and Next Steps, Wu et al, 2024) is clearly of value.<br>In general, the paper provides the key information necessary for public health researchers to understand the methodology, measures available, limitations and uses of the UKHLS, but would benefit from some amendments to focus and improve reporting on these topics.<br>Major comments<br>The organisation of the sections 'What is already known on this topic'; 'what this data resource adds' and 'how this data resource might affect research, practice or policy' could be briefer and more clearly laid out. Typically in BMJ Medicine these include 2-3 key bullet points. These sections could be improved by, for example, focusing more specifically on comparisons of the UKHLS with other longitudinal surveys, in particular with cohort or cross-sectional resources.<br>A key need for public health researchers in understanding the value and limitations of the UKHLS is the sampling methodology. This is particularly important with the UKHLS, as availability of longitudinal data on households is one of the key strengths of the dataset, which requires sampling approaches that may not be familiar to all public health researchers.<br>This is described in the paper as "an address-based probability sample to ensure the study is representative of residents in households across the UK" with a note that participants in Northern Ireland are "selected from a simple random sample". The distinction here is not entirely clear. Other papers include brief additional details on sampling design (e.g. Lynn et al, 2023; "a stratified two-stage sample, with postcode sectors selected at stage one followed by residential addresses at stage two"); this, or a comparable statement would be useful to clarify the approach. Given researchers may be selecting between datasets to answer specific research questions, it would be helpful to understand any evidence on whether this approach achieves greater or less attrition and is more or less subject to biases (e.g. in respondents who are typically excluded from survey data) than more common cohort approaches to data gathering.<br>Some further details on attrition would also be of use. Table 1 gives

some brief details on attrition (reporting how many panel members have provided reported measures for the main questionnaire over all waves and at least 3 waves). However, it is not immediately obvious what the attrition rate is over years, and if there were any differences between rates of attrition for key subgroups. Table 1 reports on the sample size for each wave, beginning with 50,866, falling to 35,262 for Wave 14, with a slight rise at Waves 6 and 14, presumably from the inclusion of the ethnic minority boost (Wave 6) and the general boost (wave 14) reported in the text. The total N is given as 97,896, alongside a figure for the number for at least 3 measures collected (60,694). It is not clear how these figures relate; has there been additional recruitment to 'top up' the sample at different years? It would be helpful to report on the attrition between waves as a percentage in Table 1 and comment in the text if there is evidence of specific exogenous factors (e.g. the COVID-19 pandemic) affecting attrition disproportionately at specific times.
Other literature referenced in the paper (Cabrera-Álvarez, James and Lynn, 2023) notes specific attrition rates for the ethnic minority boost within the UKHLS; if any analysis has been undertaken on whether this or other marginalised groups experience attrition at a different rate to the main sample, this would be useful to report.
Given that many national level surveys have been required to change their mode of collection over time (e.g. the National Survey for Wales) and that this may have led to variation in data collection, a comment on whether the collection of data via questionnaire has changed over time would be helpful, particularly in relation to data collected during the COVID-19 pandemic, which is an area many public health researchers are particularly interested in researching.
Clearly a wide range of topics are covered by the UKHLS, and the paper usefully describes a number of these. However, whilst there is considerable detail on biological data measures reported (Table 2) only a small number of other health and social indicators are mentioned and only five are noted in tabular form in Table 1. Given the importance of health behaviours and social conditions to the public health research field, presentation of a wider range of these would be appropriate in this context. It would be particularly useful to understand if there are specific questions on common measures such as 'deprivation' (as there are in some surveys, e.g. family affluence, in the Secondary School Children's Health and Well-being survey in Wales) or if these are linked to existing area level metrics such as the Index of Multiple Deprivation.
The paper is particularly useful when it highlights the differences between the UKHLS as a resource when compared with other studies. Of particular relevance to public health research are the household design, the length of time covered by the data, the range of measures captured, the ethnicity boost and the availability of biomarkers. Longitudinal sampling of households is particularly of interest, as there is a lack of large survey-based research datasets that permit looking at a representative sample of the population over time, as opposed to repeating cross-sectional surveys or following a birth cohort.
The paper usefully provides examples of studies that have used UKHLS across a range of public health topics. As public health is a broad discipline, and researchers are likely to be looking for application to specific questions, this approach might be even more effective if studies were organised by theme including examples from both topic areas (e.g. health behaviours, social determinants of health) and research designs (e.g. multilevel models, case control studies). The fact that longitudinal data that provides insights into the dynamics of early life settings is of particular value as it supports

	the testing of causal hypotheses on the influence of these domains, an area of increasing interest in public health research.<br>The inclusion of Table 4, documenting linkages with other datasets is valuable to have.<br><br>Minor comments<br>Given that health policy is devolved to the governments of Northern Ireland, Wales and Scotland, an issue for public health researchers in those nations is challenges of using survey panel data because smaller populations mean smaller sample sizes. Other papers describing the sampling strategy (e.g. Lynn et al, 2023) suggest that samples from UK nations other than England are boosted; it would be of value to public health researchers focusing on research to inform policy in these nations to be aware of this benefit.<br>In relation to references:<br>• Reference 4 (Whitley et al) lacks a DOI, which is https://10.1016/j.intell.2016.07.001<br>• Reference 5 (Brins et al) also lacks a DOI, which is: https://doi.org/10.1038/s41598-017-10812-1<br>• Reference 6 has a DOI but lacks other details (volume number, page numbers)<br>• Reference 10 is for a pre-print paper – a peer reviewed version of this paper appears now to have been published and therefore the reference should be updated: Shen X et al, A methylome-wide association study of major depression with out-of-sample case–control classification and trans-ancestry comparison, Nature Mental Health volume 3, pages1152–1167 (2025) - https://www.nature.com/articles/s44220-025-00486-4<br>• DOI links are not entirely consistent, some are presented as the DOI alone, some with URLs. It would be helpful to present all with URLs

VERSION 1 – AUTHOR RESPONSE

We thank all four reviewers for their positive comments on our manuscript. We address feedback and requests for additional information below.

Reviewer: 1

Prof. Katie Harron

What this data resource adds: This section says that for some households, data goes back to 1991. It would be helpful here to state when the main data collection began.

RESPONSE: We apologise for a lack of clarity. We have amended line 1 in section to include the start date for UKHLS. It now reads as 'UKHLS is an infrastructure resource, which since 2009 has collected data annually on household members of all ages from a sample (approximately 30,000 households;48,000 adults, 5,000 adolescents (10-15 years) and 11,000 children under 10 at wave 1) of households selected to be representative of the countries in the UK.'

Table 1 – if they are currently on wave 16/17 data collection, why does this table not include wave 15? Do the numbers in this table represent unique individuals? If so, would it be possible to give the number of households too?

RESPONSE: Final numbers for wave 15 were not available when this manuscript was submitted.

In response to this query, we have clarified units in the title of table 1 which now states 'Number of adults with data available for each measure'. We have not added the number of households with specific measures at each wave as this would double the size/complexity of the table, and we feel for this audience individual level data would be more relevant.

Could you expand a bit more on the dynamic nature of the cohort – and the number of households / individuals for whom you have data for the whole period? i.e. what is the average (and total) length of follow up within the study?

RESPONSE: Thank you. We included numbers of participants for whom we have these specific measures at all waves in table 1 as described above. Due to the limited word count for this manuscript we refer to extensive literature we publish on attrition (eg ref 2), including an annual report Cabrera-Alvarez and Lynn (2026) for the reader which we have now included in the manuscript.

Would it be possible to include some simple characteristics of those included, e.g. age / sex / region / ethnicity?

RESPONSE: We included this information in the preliminary section of the manuscript. In response to this query, we add a little more detail to Section 2. 'UKHLS is an infrastructure resource, which provides data, collected annually on household members of all ages from a sample (approximately 30,000 households;48,000 adults, 5,000 adolescents (10-15 years) and 11,000 children under 10 at wave 1) of households selected to be representative of the countries in the UK. It has oversampled the five main ethnic minority groups aimed to enable a sufficient sample size for key ethnic minority groups in the UK, and has periodic refreshment samples to ensure it maintains its sample size and representativeness more broadly.'.

Could you provide a bit more information about the involvement rates? i.e. for those who are invited, what percentage typically respond?

RESPONSE: In the 'missing data' section, we state that 'UKHLS achieves wave on wave response rates of around 90% '. Much more detail on involvements rate are available on the website updated with each wave. https://understandingsociety.ac.uk/wp-content/uploads/documentation/user-guides/6614_main_survey_user_guide_response_tables.pdf.

Reviewer: 2

Prof. Neil Davies, UCL

Section 1 – I would add genetic and epigenetic data here.

RESPONSE: Thanks for this suggestion. The sentence in section 1 is amended to ‘covers a wide range of topics, including health, biomarker, genetic and epigenetic data’

Section 3- It would be useful to provide some summary of the number of applications to use the data, or the number of papers published (if possible?)

RESPONSE: A short sentence has been added to section 3 ‘. For example, there have been over 100 publications from the genomic data.’

“Findings from the UKHLS…” This needs references.

RESPONSE: A link to the UKHLS webpage that provides an overview of the impact from research based on the dataset has been added to the line ‘Findings from UKHLS have been used widely to influence public health and social policy (see Case studies - Understanding Society).’

“Additionally, biosampling and clnical…” – would be good to summarise the ages or years when the biosampling occurred if possible here.

RESPONSE: This sentence is amended to ‘Additionally, biosampling includes measures of genetic and epigenetic markers and clinical measures are included to provide objective measures of health in adult aged over 16 years.’

One of the unique features of US is that it is a representative random sample. Are there any key results or references that demonstrates the importance of this design for getting unbiased or less biased estimates? This is a key strength relative to other studies, such as the UK Biobank, that are bigger, but potentially more biased by differential ascertainment. You do make this point, but it really needs a compelling example to demonstrate how important this is for epidemiological studies. Alterantively, do we know when this is likely to be most, and less important (e.g. genetic studies, studies of inequalities, descriptive studies)? Feel free to say these are all unknowns that need more work. Do we have any information on the relative representativeness of US vs other representative samples such as the HSE? Is there code to create weights for analytic samples?

RESPONSE: Representativeness is important when researching prevalence estimates. Further it is likely that social science or inequalities research is benefited by the availability of datasets that are not biased by the factors under investigation. The UKHLS data include weights (Weighting guidance - Understanding Society to enable the research community to make population level inferences. We have added this link to the ‘what population does UKHLS cover’? Other information is provided to enable researchers to understand the relative representativeness of UKHLS, for example a comparison of the biomarker data in UKHLS with those available in the Health Survey for England can be found in the Biomarker Glossary [Biomarker User Guide and Glossary - User Guide]. We have added this point to Box 2.

Linkage – US is also available within the LLC, might be worth flagging this in the penultimate para of page 7.

RESPONSE: Thank you. Information on data linkage and the LLC is mentioned on page 7 and in Box 1.

Genomic and Methylation data – would be worth including more details on this – how many samples, what chips, what imputation and QC have been done etc. This is another key feature of US, and would be great to include the relevant details for your readers.

RESPONSE: In order to remain within the word count limit, additional citations to manuscripts with more detailed information on imputation and QC has been added to the legend in table 2. The Illumina Infinium HumanCoreExome BeadChip Kit® (12v1-0) was used for genotyping and the Illumina 'EPIC' array for the measurement of methylation are also mentioned in table 2.

Boxes 1 and 2 are useful – for typesetting it would be good to make sure the links are well formatted on the website etc.

Would it be worth including adding the genomics and methylation data to the LLC in the future plans?

Would it be possible to create a graph that summarises the data collection in US? For example, we used the following figure in our recent IJE profile on the genomics resources for the CLS cohorts (Figure 1):

https://academic.oup.com/ije/article/54/5/dyaf141/8237599

RESPONSE: we thank you for this suggestion. However, a figure such as the one suggested would not be useful as UKHLS is an all age panel study that strives to maintain year on year response rate and periodically boosts sample to maintain representativeness and thus year on year numbers are largely static.

Are there any resources e.g. code for QC and data cleaning? Would it be worth flagging any of the work by CLOSER etc (totally optional, a reasonable response is "no".

RESPONSE: A number of resources are available to support users of UKHLS. A link to our syntax page is provided in box 2.

Reviewer: 3

Dr. Nazrul Islam, University of Southampton

"The fundamental design of UKHLS is an address-based…" does it mean postcode based?

RESPONSE: Apologies if this is confusing. 'Address based' means the design is a stratified clustered sample of postcode sectors from which addresses are randomly selected.

"What does the UKHLS measure?" section says it has four types of data— I would suggest using sub-headlines with exact four types (currently more than four subsections).

RESPONSE: We apologise that there was a typo in this section and we have amended the number of types of data from four to five.

“Measures of cognitive performance were collected at wave 3 (Table 3)…” It is not clear which cognitive measures are being referred to; table 3 only says “Cognition – 9 items from Ravens Standard Progressive Matrices (wave 10 only)”.

RESPONSE: Table 2 lists the study’s measures of memory and executive function measured in wave 3 (2011-2012) in all adults (aged 16+). Table 3 lists the measures of cognition captured in youth (aged 10-14) in wave 10 (2018-2019).

“…confirm previously observed patterns of cognitive aging.” Not sure what this is referring to.

RESPONSE: We refer to reference 4 Whitley et al., 2016. The paper described the associations between the measures of cognition collected in the study and how they differed across the entire lifespan.

“Genomic data on a sub-sample of those who took part in the nurse interview and consented to DNA extraction.” Could the authors provide a bit more information on the response rate, any sampling bias, and any proposed measures to make them representative? If not, could be included as a limitation.

RESPONSE: Currently genomic data are available from White/European participants only. We are creating weights for these subsets of data and these will be available soon. Further, the study is currently in field (Wave 16) and will supplement the genomic datasets with a wider range of participants, including non-White/European groups and those participants that have become adults since waves2/3.

“Participants have also been asked to give consent to linkage of their data to routine health and other administrative data, with ongoing linkage processes (Table 4).” First, this should not be included under four types of data; maybe elsewhere. Second, could the authors clarify which datasets (primary care, secondary care etc) are these referring to?

RESPONSE: Table 4 now clarifies that we are linking to datasets that include Hospital Episode statistics

A table showing the baseline characteristics of the original wave, most recent wave, and national population would be helpful to show representativeness.

RESPONSE: The UKHLS team provides a number of publications that address the representativeness of the dataset. As we are limited by a word and reference count, we refer you to reference 19, which describes the representativeness of the sample at Wave 15 (2023-2025).

A visual flow diagram showing how many participants continued to remain in the cohort, and how many participants were later included (and whether they remained or lost to follow-up) including their children could be very informative.

RESPONSE: we thank you for this suggestion, however, such a flow diagram is likely to be uninformative, given the complex design of UKHLS. The study is composed of a number of sub groups – the general population sample, the on-going British Household Panel study, the ethnic minority boosts and additional boosts to maintain representativeness in the study. Further as stated in the section 'What UKHLS includes', people join and leave and can return to the study at each wave– thus we state 'People who join an 'UKHLS' household, for examples as partners, new babies, or lodgers are temporarily included in the study while in the household. If they have a child with an original sample member, or are born to a sample member, they become study members and are followed if they leave the original home. Any original sample member who splits off from their original household is followed, and the people they form a new household with become part of the study as above. We have therefore decided against the inclusion of a visual flow diagram in our submission.

Minor: Some sources are referred to as hyperlinks. Perhaps better to use as proper sources using proper format?

RESPONSE: the format for this is submission is limited by a tight word and reference count and we therefore cannot add to the reference list. The hyperlinks enable the reader to readily access additional information if they so wish.

Reviewer: 4

Mr. Chris Emmerson, Public Health Wales

Major comments

The organisation of the sections 'What is already known on this topic'; 'what this data resource adds' and 'how this data resource might affect research, practice or policy' could be briefer and more clearly laid out. Typically in BMJ Medicine these include 2-3 key bullet points. These sections could be improved by, for example, focusing more specifically on comparisons of the UKHLS with other longitudinal surveys, in particular with cohort or cross-sectional resources.

RESPONSE: In the section on what this data resource adds, we provide a number of features of UKHLS that are not available in other resources. The word limit precludes from a more explicit comparison of UKHLS and other studies.

A key need for public health researchers in understanding the value and limitations of the UKHLS is the sampling methodology. This is particularly important with the UKHLS, as availability of longitudinal data on households is one of the key strengths of the dataset, which requires sampling approaches that may not be familiar to all public health researchers.

This is described in the paper as "an address-based probability sample to ensure the study is representative of residents in households across the UK" with a note that participants in Northern Ireland are "selected from a simple random sample". The distinction here is not entirely clear. Other papers include brief additional details on sampling design (e.g. Lynn et al, 2023; "a stratified two-stage sample, with postcode sectors selected at stage one followed by residential addresses at stage two"); this, or a comparable statement would be useful to clarify the approach. Given researchers may be selecting between datasets to answer specific research questions, it would be helpful to understand any evidence on whether this approach achieves greater or less attrition and is more or

less subject to biases (e.g. in respondents who are typically excluded from survey data) than more common cohort approaches to data gathering.

RESPONSE: Thank you. We discuss the sampling strategy above regarding the addressed based approach in the study design (reviewer 3 comment 1). The sampling strategy of UKHLS enables us to create and provide weights for the research community. As mentioned in 'What population does UKHLS cover?' it is possible to assess biases created through non-response and attrition and re-weight the sample back to be representative of the UK at baseline. This enables the production of disease and other prevalence, not possible for volunteer and other studies. The effort taken by the study to ensure that a variety of groups respond to the survey enables analyses not possible in other studies.

Some further details on attrition would also be of use. Table 1 gives some brief details on attrition (reporting how many panel members have provided reported measures for the main questionnaire over all waves and at least 3 waves). However, it is not immediately obvious what the attrition rate is over years, and if there were any differences between rates of attrition for key subgroups. Table 1 reports on the sample size for each wave, beginning with 50,866, falling to 35,262 for Wave 14, with a slight rise at Waves 6 and 14, presumably from the inclusion of the ethnic minority boost (Wave 6) and the general boost (wave 14) reported in the text. The total N is given as 97,896, alongside a figure for the number for at least 3 measures collected (60,694). It is not clear how these figures relate; has there been additional recruitment to 'top up' the sample at different years? It would be helpful to report on the attrition between waves as a percentage in Table 1 and comment in the text if there is evidence of specific exogenous factors (e.g. the COVID-19 pandemic) affecting attrition disproportionately at specific times. Other literature referenced in the paper (Cabrera-Álvarez, James and Lynn, 2023) notes specific attrition rates for the ethnic minority boost within the UKHLS; if any analysis has been undertaken on whether this or other marginalised groups experience attrition at a different rate to the main sample, this would be useful to report.

RESPONSE: There are a number of publications that address the issue of attrition within the study, including an annual report (ref 19 is the latest). Because of the word count limit for this submission, we could not discuss attrition in the detail that is being requested. We cite a number of outputs from the study, reference 2 and reference 19, as noted.

Given that many national level surveys have been required to change their mode of collection over time (e.g. the National Survey for Wales) and that this may have led to variation in data collection, a comment on whether the collection of data via questionnaire has changed over time would be helpful, particularly in relation to data collected during the COVID-19 pandemic, which is an area many public health researchers are particularly interested in researching.

RESPONSE. We thank the reviewer for this feedback. We have added a sentence in paragraph 1 in 'What does UKHLS measure' to address this comment 'From waves 1-7 Understanding Society was a face-to-face survey, with minor mop-up survey by telephone. From waves 8, web data collection was also introduced, and the study became a mixed mode survey. This meant that when the COVID-19 pandemic started, the study was able to continue without any loss of fieldwork'. The UKHLS team are experts in the area of survey methodology and have published a number of working papers describing the impact of mode and other methodological issues on participation and measurement. These can be found here: Understanding Society Working Paper Series Archives - Understanding Society

Clearly a wide range of topics are covered by the UKHLS, and the paper usefully describes a number of these. However, whilst there is considerable detail on biological data measures reported (Table 2) only a small number of other health and social indicators are mentioned and only five are noted in tabular form in Table 1. Given the importance of health behaviours and social conditions to the public health research field, presentation of a wider range of these would be appropriate in this context. It would be particularly useful to understand if there are specific questions on common measures such as 'deprivation' (as there are in some surveys, e.g. family affluence, in the Secondary School Children's Health and Well-being survey in Wales) or if these are linked to existing area level metrics such as the Index of Multiple Deprivation.

RESPONSE: We thank the reviewer for this comment and have added a link to a more detailed content plan available on our website and added a sentence to paragraph 2 in 'What does UKHLS measure' As well as a wider range of geographic identifiers allowing linkage to contextual information such as the index of Multiple Deprivation (also provided by the study)'.

Minor comments

Given that health policy is devolved to the governments of Northern Ireland, Wales and Scotland, an issue for public health researchers in those nations is challenges of using survey panel data because smaller populations mean smaller sample sizes. Other papers describing the sampling strategy (e.g. Lynn et al, 2023) suggest that samples from UK nations other than England are boosted; it would be of value to public health researchers focusing on research to inform policy in these nations to be aware of this benefit.

RESPONSE: As a point of clarification we should note that numbers from UK nations are boosted in the predecessor of UKHLS, the British Household Panel Study. We are currently planning a country specific boost in UKHLS, which should be available in future waves.

In relation to references:

• Reference 4 (Whitley et al) lacks a DOI, which is https://10.1016/j.intell.2016.07.001

• Reference 5 (Brins et al) also lacks a DOI, which is: https://doi.org/10.1038/s41598-017-10812-1

• Reference 6 has a DOI but lacks other details (volume number, page numbers)

• Reference 10 is for a pre-print paper – a peer reviewed version of this paper appears now to have been published and therefore the reference should be updated: Shen X et al, A methylome-wide association study of major depression with out-of-sample case–control classification and trans-ancestry comparison, Nature Mental Health volume 3, pages1152–1167 (2025) - https://www.nature.com/articles/s44220-025-00486-4

• DOI links are not entirely consistent, some are presented as the DOI alone, some with URLs. It would be helpful to present all with URLs

RESPONSE: Thanks for reading our manuscript with such care. We have fixed all the issues identified.
